# Wild Blueberries (*Vaccinium myrtillus)* Alleviate Inflammation and Hypertension Associated with Developing Obesity in Mice Fed with a High-Fat Diet

**DOI:** 10.1371/journal.pone.0114790

**Published:** 2014-12-12

**Authors:** Otto T. Mykkänen, Anne Huotari, Karl-Heinz Herzig, Thomas W. Dunlop, Hannu Mykkänen, Pirkka V. Kirjavainen

**Affiliations:** 1 Institute of Public Health and Clinical Nutrition, Department of Clinical Nutrition, Food and Health Research Centre, University of Eastern Finland, Kuopio, Finland; 2 Institute of Biomedicine, University of Eastern Finland, Kuopio, Finland; 3 Institute of Biomedicine and Biocenter of Oulu, University of Oulu, Medical Center Oulu and Oulu University Hospital, Oulu, Finland; 4 Department of Environmental Health, National Institute for Health and Welfare, Kuopio, Finland; University of East Anglia, United Kingdom

## Abstract

**Background:**

Low-grade metabolic inflammation and hypertension are primary mechanisms involved in obesity-associated adverse health effects. Berries, especially Nordic wild blueberries (hereafter referred to as bilberries), represent an important source of dietary anthocyanins, a group of polyphenols with potential beneficial effects to combat obesity-associated metabolic disturbances.

**Methods:**

The effects of 5% or 10% (w/w) of whole bilberries (BB) were studied on the development of obesity and its metabolic disturbances in C57BL mice fed with a high-fat diet (HFD) for three months. Cytokines, inflammatory cells, systolic blood pressure, glucose tolerance, insulin sensitivity, weight gain, body fat, food consumption and energy metabolism were assessed.

**Results:**

Bilberries ameliorated type 1 pro-inflammatory responsiveness induced by HFD. This was indicated by the altered cytokine profile and the reduced prevalence of interferon gamma -producing T-cells, in particular T helper type 1 cells. Bilberries also prevented the progression of obesity associated long term increase in systolic blood pressure in mice.

**Conclusions:**

Bilberries reduce the development of systemic inflammation and prevent the progression of chronic hypertension, thus supporting their potential role in alleviating the adverse health effects associated with developing obesity.

## Introduction

Obesity is associated with several comorbidities, e.g. metabolic syndrome (MetS), type 2 diabetes (T2D) and cardiovascular diseases, and has also been linked to low-grade inflammation and hypertension [1], [2]. Chronic low-grade inflammation can be considered as one of the primary mechanisms behind the adverse health effects of obesity [3]–[5]. Berries, especially bilberries and blueberries have been shown to have potential in the preventive management of these complications. Their effects have been suggested to be mediated via anti-inflammatory action [6]–[9]. However, the characterization of the immunological effects of bilberries is still poorly understood and, virtually nothing is known about their systemic effects at the cellular level. The inflammatory mechanisms caused by obesity are highly complex involving several cellular components and mediators [1], [10]. Prolonged feeding of C57BL “black” mice with a high-fat diet (HFD) is known to increase adiposity and the production of inflammatory cytokines, such as tumor necrosis factor alpha (TNF-α), monocyte chemoattractant protein 1 (MCP-1) and interleukin 1-beta (IL-1β) [5]. Infiltration of cytotoxic T cells (Tc, CD8^+^ T cells) or natural killer T cells (NKT cells) into adipose tissue is mainly responsible for macrophage recruitment and the recruitment is further induced by the reduction of adaptive immune functions of T cells and regulatory T cells (Treg cells) [11], [12]. Also the systemic T helper type 1 (Th1)/Th2 cell ratio appears higher for obese animals. Th1 cells secrete cytokines, which stimulate cell-mediated immune responses central in the innate pro-inflammatory responses associated with low-grade inflammation in obese mice [13].

The C57BL mice fed with HFD become obese within weeks and with prolonged feeding they develop insulin resistance and glucose intolerance, hypertension and low grade (metabolic, meta-) inflammation [4], [5], [14]–[17]. Adipose tissue derived cytokines, especially the chemoattractant proteins, recruit lymphocytes to the site of chronic inflammation [5]. Previous studies which examined the anti-inflammatory effects of whole blueberries and bilberries of *Vaccinium* species have concluded that the health effects of these berries could be immunologically mediated [6]–[9] but, the characterization of the immunological effects is still poorly understood and little is known about their systemic effects at cellular level.

Bilberries (wild European blueberries, *Vaccinium myrtillus*) are rich in anthocyanins (ANCs), making them a unique dietary source of flavonoids [18]–[20]. Whilst the fruits and berry peels [18] are rich in ANCs, bilberries contain also phenolic compounds other than flavonoids (flavonols, phenolic acids and pro-anthocyanidins) and vitamins C and E which may also be partly responsible for their activities. Investigations examining the health benefits of berries and especially ANCs have revealed antioxidative, anti-inflammatory, antimicrobial, antidiabetic and anticarcinogenic properties [21], [22]. Bilberry fruits and leaves have been used to control glucose levels in traditional medicine but with varying success [23], [24]. Blueberries, bilberries or pure ANCs or their extracts have shown anti-obesity effects in mouse models or reduced obesity-associated symptoms [6], [25]–[27], but the findings with whole berries seem not to be as consistent as with extracts [28], [29]. Earlier studies have also shown that ANCs or their sources, other than whole bilberries, reduce blood pressure in rodent models of hypertension [30]–[32]. However, the HFD mouse model with whole bilberries (*Vaccinium myrtillus*) has only been utilized in a previous study focusing on retinal gene expression [33].

This is the first study to have utilized the HFD mouse model to comprehensively examine the effects of bilberries on the key features of MetS and obesity (energy and glucose metabolism, blood pressure and body fat content) together with an extensive concomitant analysis of their immunomodulatory effects.

## Methods

### Animal study design and diets

The diets used in this study were normal control diet (NCD, 10% kcal fat, D12450B), high-fat diet (HFD, 45% kcal fat, D12451), and 5% or 10% (w/w) of whole freeze-dried bilberries (BB) in HFD (HFD+5% BB or HFD+10% BB) (Research Diets, New Brunswick, NJ, USA. Research Diets Inc. www.researchdiets.com). This model utilizes an open source diet having a well-controlled composition and a C57BL/6J inbred mouse strain that is known to display genetic predisposition to weight gain [15], [16]. The pilot experiment using graded levels of freeze-dried bilberries enriched with small berries and peels (0–10%w/w) in HFD indicated that 5% (w/w) of freeze-dried bilberries in the diet was effective to increase insulin sensitivity at 7 weeks (Figure S1 in S1 Panel, supporting phenotypic characteristics).The 5% BB diet was matched with the control HFD for carbohydrate (sucrose and cellulose) and potassium (potassium citrate) contents and pelleted by the manufacturer. The 10% BB in HFD diet was prepared in our laboratory by replacing 10% (w/w) of HFD with freeze-dried BB and adding 13% (w/w) Milli Q -purified water for pelleting and then drying the pellets at +34°C overnight. The ACN content of the diets and the freeze-dried bilberries was analyzed with the HPLC method of Koponen et al. (2007) [19]. The energy and principle nutrient composition of the diets is shown in Table 1. Also the detailed ingredients as well as anthocyanidins (ANC aglycones), salt and lipid contents of the diets are described in the supporting information (S1 Table).

**Table 1:** Energy nutrient composition of the diets.

	NCD		HFD		5%BB in HFD		10%BB in HFD	
	(g)	(% kcal)	(g)	(% kcal)	(g)	(% kcal)	(g)	(% kcal)
**Protein**	19.2	20	23.7	20	23.5	21	21.2	19
**Carbohydrates**	67.3	70	41.4	35	39.9	33	41.8	38
**Fat**	4.3	10	23.6	45	23.5	46	21.2	43
**Total**	90.8	100	88.7	100	86.9	100	84.2	100
**Energy (kcal/g)**	3.85		4.73		4.70		4.56	

The composition of normal control diet (NCD), high-fat diet (HFD), 5% and 10% BB in HFD.

**Figure 1:**
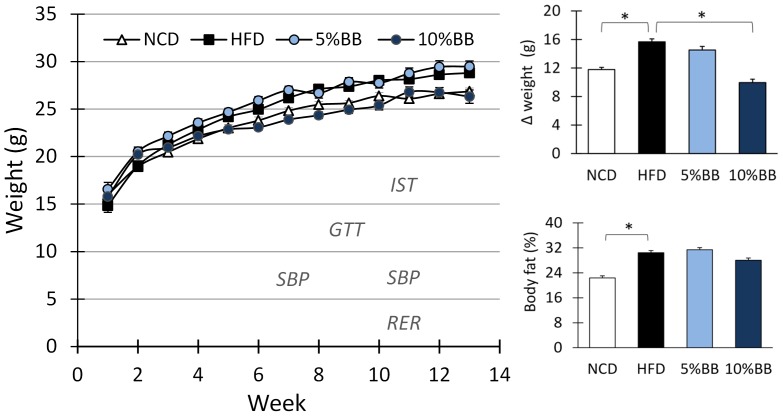
Weight gain and fat percentage of mice fed HFD and bilberries. The diets used were normal control diet (NCD), high-fat diet (HFD), 5% and 10% (w/w) BB in HFD fed for 12–14 weeks. The time points for testing systolic blood pressure (SBP), glucose tolerance (GTT), insulin sensitivity (IST) and respiratory exchange ratio (RER) in four repeated experiments are displayed in the figure. The bars represent the means and SEM of n = 22–37 mice per diet group in measured weight gain and n = 8–9 mice in measured fat %. Asterisk (*) indicates the significant differences of HFD group from NCD and 5% BB and 10% BB in HFD groups (p<0.05, Mann Whitney U-Test with Bonferroni's correction).

The diets were started at the age in 4–5 weeks and fed *ad libitum* for 3 months. Up to 200 mice (C57BL/6J) were used in four repeated sets of feeding experiments (batch) over the course of the study. The animals were randomly divided into diet groups with a maximum of 60 mice in each batch. Body weights were measured weekly and food and water consumption at weeks 1–2 (beginning), 5–7 (middle) and 11–12 (end). In addition, food and water intakes were assessed during weeks 8–12 (end) with an automated metabolic phenotyping unit (LabMaster; TSE Systems, GmbH, Bad Homburg, Germany) [34]. After 12 weeks of feeding the animals were euthanized using CO_2_ asphyxiation and cervical dislocation and blood samples were collected for glucose, insulin, lipid and cytokine measurements. The other measured phenotypic parameters included glucose tolerance (GT), insulin resistance (IR), systolic blood pressure (SBP), weight gain, respiratory exchange ratio (RER) and body fat %, the time points of measurements are shown in Figure 1. Systolic blood pressure was measured with a Tail Cuffs System and fat disposition using magnetic resonance imaging (MRI) at 12 weeks. All procedures were performed according to the national legislation on animal experimentation and approved by the Committee of Animal Experiments in the County Administrative Board (The State Provincial Office of Finland, Hämeenlinna, Finland).

### Energy metabolism, locomotor activity, and body fat percentage

HFD consumption has been associated with increased and uncontrolled energy intake and reduced activity [35]. To examine the effects of HFD and bilberries on energy expenditure, a calorimetry system (LabMaster) was used to measure locomotor activity, RER and lipid utilization. The instrument consists of a combination of sensitive feeding and drinking sensors for automated online measurements. The calorimetric system is an open-circuit system that determines continuously and simultaneously O_2_ consumption, CO_2_ production, and therefore RER of the estimated respiratory quotient (RQ  =  *V*CO_2_/*V*O_2_). The laser beam-based activity monitoring system detects and records ambulatory movements, including z-axis events such as rearing and climbing. The mice were placed in cages after a 3–5 day training period using similar drinking bottles and cages in the same room (run-in phase). After adaptation the data was collected over a 3–4 days per mouse. A total of 6 mice per diet group (4 diet groups per experiment) were recorded using mice from two repeated experiments. For the determination of body fat content, mice were anesthetized with isoflurane and the body fat was measured at 12 weeks from eight mice per group with MRI with nitrogen/oxygen (70/30, vol/vol) carrier and 4.7T Magnex magnet combined with a Varian UNITY INOVA console [36]. All data from the T1-weighted images were gathered as 3D-gradient echo images with a 0.39x0.31x0.31mm^3^ resolution and analyzed with Matlab from neck to the basal tail to gain total body fat %.

### Glucose tolerance and insulin sensitivity

Glucose tolerance and insulin sensitivity testing were carried out using a previously described protocol [37]. In brief, the intraperitoneal glucose tolerance test (IPGTT) was performed at 9 weeks by administering i.p. 10 µl/g (weight) of 20% (w/v) glucose in 0.9% NaCl (2 g/kg weight of mice). The blood glucose concentration was measured from the tail vein after 12–14 h (overnight) fasting at baseline (0 min) and 15, 30, 60, 90 and 120 min after i.p. glucose administration using One Touch Ultra analyzer (LifeScan, Milpitas, USA). Glucose tolerance was measured in each experiment using a total of 20–28 mice per group.

Insulin sensitivity was measured at 11–12 weeks with i.p. administration 10 µl/g (weight) of 0.025 IU/ml Humulin insulin in 0.1% bovine serum albumin (w/v) and 0.9% NaCl (w/v) (0.25 IU/kg weight of mice). Blood glucose was analyzed from the tail vein with the One Touch Ultra Analyzer after a 2-hour fast at baseline (0 min) and at 15, 30 and 60 min after i.p. injection. Since fasting glucose levels differed between the groups, the percentage values were calculated to examine the relative insulin sensitivity [37]. Insulin sensitivity was measured in each experiment using a total of approximately 20 mice per diet group. The area under the curve (AUC) was analyzed using Graph Pad Prism software.

### Serum lipids, cytokines and insulin

Blood serum was collected using Microvette CB 300 capillary tubes according to the manufacturer (Sartsedt AG & Co, Nümbrecht, Germany) from 15–20 mice/diet group when sacrificed for the analysis of serum lipids (triglycerides, HDL, LDL, total cholesterol and non-esterified free fatty acids/FFA) using the Thermo Fisher Konelab 20XTi Analyzer (Thermo Electron Corporation, Vantaa, Finland).

Cytokines and adipokines were analysed from blood serum or heparinized plasma samples (10–20 mice/diet group) using the LINCOplex ELISA multi analyte detection system applied in Luminex instrumentation (Linco Research Inc, USA). The cytokines; MIP-1α, G-CSF, MCP-1, KC, RANTES, IFN-γ, IL-1β, IL-1α, GM-CSF, IP-10, IL-2, IL-4, IL-5, IL-6, IL-7, IL-10, IL-12(p70), TNF-α, IL-9, IL-13, IL-15, IL-17 were measured in samples diluted 2∶3 in the serum matrix using the mouse Multiplex kit (Lincoplex KIT MCYTO 70K, Linco Research, St. Charles, Missouri, U.S.A.). Quantification was conducted using the best fit equation of standards (2-2000 pg/mL) in Curve Expert software with correlation coefficients above 0.999 (http://www.curveexpert.net). For analysis of adiponectin, insulin, leptin, resistin and tPAI the samples were diluted 1∶4 in serum matrix (MADPK-71K, Millipore Corporation, Billerica, MA, USA). Measurements were carried out in Bio-Rad Bead Station (v.4.1.1, Biorad, Hercules, USA) according to the manufacturer's instructions. Data analysis was carried out with a logistic 5PL fit and the quality of runs was ensured by daily re-calibration and monitoring CV% of the standards and the provided controls, bead counts and aggregates. Samples with a high percentage of aggregates or multiple sampling errors were excluded. Further analysis of the net effects of grouped cytokines on potentially pro-inflammatory and Th1 responses were done by calculating the sum of z-scores of the respective cytokines. Grouping was carried out according to the cytokine functions [38]–[40]: the most common pro-inflammatory cytokines (IL-1α, IL-1β, IL-2, IL-6, IL-12, IL-15, IL-17, RANTES and TNF-α), the obesity associated pro-inflammatory cytokines (MCP-1, IL-1β, IL-6 and TNF-α), and the T helper cell type 1 cytokines (IL-2, IL-12, INF-γ and TNF-α).

### Cellular markers of inflammation

Lymphocytes were instantly harvested from the spleens of euthanized mice as previously described (n = 6–11 per group) [41]. The relative presence of different lymphocyte populations and their intracellular cytokine production were determined by flow cytometer [42]. For the detection of intracellular cytokines, the cell preparations were stimulated for 5h at 37°C with 5% CO_2_ with brefeldin A at 10 µg/ml, phorbol 12-myristate 13-acetate (25 ng/µl) and ionomycin (1 µg/ml) (Sigma, St. Louis, MO, USA). Initially the cells were incubated with Mouse BD Fc Block (BD Biosciences, San Diego, CA) to reduce non-specific binding and then stained with surface marker specific fluorochrome-conjugated antibodies (anti-CD3-AlexaFluor(AF) 700 (clone 17A2), anti-CD4-APC-Cy7 (RM4-5), anti-CD8a-PE-Cy7 (53–6.7) (BioLegend, San Diego, CA, USA), and anti-NK-1.1-PerCP-Cy5.5 (PK136) (BD Biosciences) followed by fixation. The cells were subsequently permeabilized and stained according to the manufacturer's instructions for cytokine specific antibodies for IFN-γ, IL-4 and IL-17 (anti-IFN-γ-AF488 (XMG1.2), anti-IL-4-PE (11B11) and anti-IL-17-AF647 (TC11-18H10.1), BioLegend, San Diego, CA, USA) utilizing the FIX&PERM reagents (Caltag Laboratories, Invitrogen, UK). Data acquisition was performed with a flow cytometer (BD FACSCanto II) and data analysis using BD FACSDiva software (BD Biosciences, San Diego, CA).

### Systolic blood pressure

Blood pressure and heart rate were recorded with a non-invasive indirect method using photoelectrical detection of blood flow in Tail Cuffs System (IITC Life Science Inc. CA, USA) with slight modifications from the previously described protocol [43]. Mice were adapted to the measuring conditions with daily 5–15 min training period for 1–2 weeks. The systolic blood pressure (SBP) was measured in restrainers after a short adaptation and sham measurements (3–5 min) followed by 10 repetitive measurements at 33°C during the same daily hours. The heart rate and blood pressure were estimated from a minimum of six repeated measurements.

### Statistical analysis

The statistical testing of the results was performed using the SPSS software (IBM Corp, Version 19.0, Armonk, NY, USA). The differences between the groups were compared with the nonparametric tests (Kruskal-Wallis test followed by Mann-Whitney U-test) and the normally distributed data with the one-way ANOVA, all tests being subjected to the Bonferroni's correction for multiple testing to avoid false positives. Correlation analyses were carried out using a Spearman's bivariate method. The values for HFD group were compared to the values for NCD group and to those of the HFD groups containing 5% or 10% BB. All data were presented as mean ± standard error of the mean (SEM) or standard deviation (SD). The data visualization and exportation was carried out using Microsoft Excel (v. 14.0.7106.5003) applied with Daniel's XL Toolbox (v.6.22) in Office surroundings.

## Results

### Weight gain, body fat percentage, feed consumption and energy metabolism

Mice fed with HFD gained more weight and had increased body fat % as compared to mice fed with NCD (p<0.05) (Figure 1). The locomotor activities and heat production remained the same between the groups (data not shown), but mice in HFD consumed significantly less food throughout the entire study period than mice in NCD (Table 2 and Table S2). The mean energy intakes however did not differ between the diet groups, due to the higher energy density (Table 1 and 2). In addition, mice on the HFD consumed less carbohydrate and more fat than those on the NCD, which was also verified by the respiratory exchange rate (RER: NCD 0.93±0.1 and HFD 0.81±0.1, p<0.05). The average anthocyanin intake in mice fed with bilberries was 1.9 mg/day and 7.0 mg/day in diet groups receiving 5% and 10% BB, respectively.

**Table 2:** Food consumption and calculated intake of macronutrients.

	NCD	HFD	5%BB in HFD	10%BB in HFD
**Food (g/d)**	3.0±0.1	2.4±0.1^a^	2.7±0.1	2.2±0.1
**Food (mg/g/d)**	113.0±2.7	86.4±2.4	92.4±4.2	84.8±3.3
**Protein (mg/g/d)**	21.5±0.5	20.5±0.6	21.7±1.0	18.0±0.7
**Carbohydrates (mg/g/d)**	75.4±1.8	35.0±1.0^a^	36.9±1.7	35.4±1.4
**Fat (mg/g/d)**	4.9±0.1	20.4±0.6^a^	21.7±1.0	18.0±0.7
**NaCl (mg/g/d)**	0.28±0.01	0.26±0.01	0.27±0.01	0.23±0.01
**Cholesterol (**µ**g/g/d)**	0.12±0.00	0.11±0.00	0.11±0.01	0.10±0.00
**Energy (kcal/g/d)**	0.43±0.01	0.40±0.01	0.43±0.02	± 0.02

a) Significantly different as compared to NCD (p<0.001, Mann Whitney U-Test with Bonferroni's correction).

Mice were fed with NCD, HFD and HFD with 5 or 10% BB for three months. Values are daily intakes measured at weeks 11–12, adjusted for weight, and presented as the mean ± SEM of n = 20–26 mice per diet group (see details in Table S2).

### Glucose tolerance and insulin resistance

Mice on HFD displayed a significantly higher AUC_glucose_ in GTT (became glucose intolerant) as compared to mice on NCD, whilst the mice on 5 or 10% BB in HFD did not differ from those receiving HFD (Figure S3 in S1 Panel, supporting phenotypic characteristics). The fasting glucose levels in IST (2 h fast) and GTT (12–14 h fast) were significantly higher in mice having HFD than in mice on NCD (NCD; 8.4±0.3 and 5.1±0.4 mmol/l, HFD; 9.7±0.3 and 6.0±0.3 mmol/l, p<0.05) (Figures S2 and S3 in S1 Panel, supporting phenotypic characteristics). The AUC_glucose_ in the IST was also significantly higher in mice fed HFD with or without bilberries than in those fed with NCD, indicating that mice on HFD became more insulin resistant and bilberries could not prevent it (Figure S2). The relative insulin sensitivity did not differ between the diet groups. Nevertheless, the glucose levels at sacrifice were significantly lower in mice fed with NCD or 10% BB in HFD (9.5±0.6 or 9.3±0.5 mmol/l) as compared to mice fed HFD alone (10.7±0.4 mmol/l). Also, the levels of insulin at sacrifice were significantly lower in NCD mice (343±81 pg/ml) than in HFD mice (835±168 pg/ml), but were not affected by the addition of bilberries to HFD (5% BB: 828±137 pg/ml; 10% BB: 946±138 pg/ml).

### Serum lipid profiles and adipokines

The total cholesterol levels were significantly increased in mice fed HFD and 5 or 10% BB in HFD (3.4±0.2, 3.9±0.2, 3.9±0.2 mmol/l, respectively) as compared to mice fed NCD (2.5±0.1 mmol/l) (p<0.05). The increase was mainly due to an increase in the HDL cholesterol concentration. HFD alone, but not with bilberries (10%), tended to increase FFAs (Table S3 in S1 Panel, supporting phenotypic characteristics). HFD significantly increased, whilst bilberries had no marked effect on leptin levels (Table 3). The resistin levels were significantly reduced in 10% BB in HFD mice, while those of adiponectin, a known anti-inflammatory adipokine, showed a decreasing trend in HFD and an increasing trend in BB supplemented HFD (Table 3).

**Table 3:** Serum levels of adipokines in mice fed with HFD or HFD with bilberries.

Adipokines	NCD	HFD	5%BB in HFD	10%BB in HFD
**Leptin (ng/ml)**	2.8±0.3	**6.1±1.2**	6.1±1.9	5.6±1.0
**Resistin (ng/ml)**	2.3±0,2	2.0±0.8	1.8±0.2	1.3±0.2^a^
**Adiponectin (µg/ml)**	26.1±3.0	20.6±1.5	22.0±2.1	28.8±6.0

In bold the trend for difference as compared to NCD (p<0.1, Mann Whitney U-Test with Bonferroni's correction).

a) Significantly different as compared to HFD (p<0.05, Mann Whitney U-Test with Bonferroni's correction).

The mice were fed with normal control diet (NCD), high-fat diet (HFD) and 5% or 10% BB in HFD for 12–14 weeks. Values are means ± SEM of n = 4–10 mice per diet group.

### Cytokines, hormones and cellular markers of inflammation

**Figure 2:**
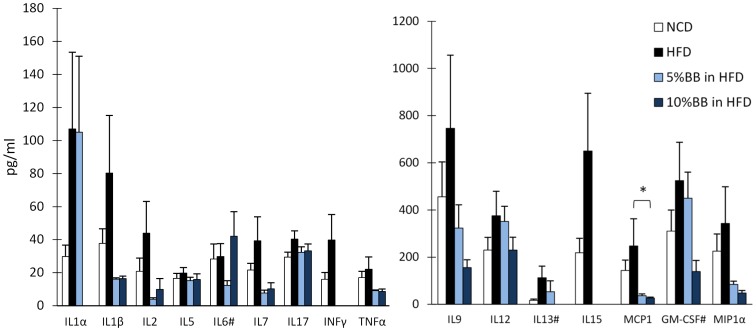
Serum levels of pro-inflammatory cytokines in mice fed with NCD, HFD or HFD with bilberries. The bars represent the means and SEM of n = 3–15 mice per diet group. The pleiotropic cytokines with both pro- and anti-inflammatory actions are marked with a number sign (#). Asterisk (*) indicates the significant differences of HFD group from 5% BB and 10% BB in HFD groups (p<0.05, Mann Whitney U-Test with Bonferroni's correction).

**Figure 3:**
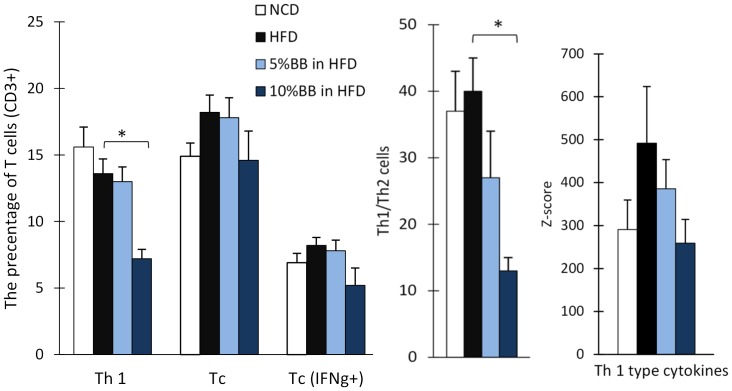
Proportion of pro-inflammatory cells and levels of cytokines in serum from mice fed NCD, HFD or bilberries in HFD. T helper type 1 cells (Th 1, CD3+CD4+IFNg+IL4-), cytotoxic T cells (Tc, CD3+CD8+) and Tc secreting INFg as precentage of T cells (CD3+) in spleen. The bars represent the mean and SEM of n = 6–11 mice per diet group. Asterisc (*) indicates the significant differences of HFD group from NCD and 5% BB and 10% BB in HFD groups (p<0.05, Mann Whitney U-Test with Bonferroni's correction).

The levels of pro-inflammatory cytokines, chemokines and growth factors are shown in Figure 2 and Table S4 in S1 Panel, supporting phenotypic characteristics. These levels were higher in HFD compared to NCD fed mice, but only with interleukin 13 (IL-13) the trend approached statistical significance (p<0.1). In contrast, the levels of several pro-inflammatory cytokines (IL-1β, IL-2, IL-7 TNF-α, GM-CSF and MCP-1) tended to be lower with the BB supplemented diets. From these cytokines, MCP-1 was significantly reduced with both BB diets. G-CSF tended to increase with BB feeding. Since the detection of several cytokines, i.e. IL-1α, IFN-γ, IL-13 and IL-15, was limited in mice with diets containing BB, we summarized the changes in functional categories of cytokines using the z-scores (Figure 3 and Figure S4 in S1 Panel, supporting phenotypic characteristics). The pro-inflammatory cytokines showed a mild increase after HFD vs. NCD (p<0.2), and the addition of BB to HFD seemed to prevent dose-dependently this increase in pro-inflammatory (Figure S4 in S1 Panel, supporting phenotypic characteristics) and T helper type 1 cytokines (Figure 3). Accordingly, the number of type 1 cells of all T cells was significantly reduced in 10% BB fed mice. Cytotoxic T cells and the ratio of Th1 to Th2 cells also showed a dose dependent reduction in BB fed mice (Figure 3). The percentage of NKT cells, mature NKT cells, INF-γ producing cells of all T cells, Th1/Th2 ratio and Th1 cells were all reduced in mice having 10% BB in HFD as compared to HFD alone (Table 4). In addition, the percentage of NKT of all cytotoxic cells was significantly lower in 10% BB in HFD than in HFD only (2.5±0.2 vs.7.0±1.2, p<0.05).

**Table 4:** Cellular markers of inflammation in mice after feeding with NCD, HFD or HFD with bilberries.

Cell Type	Label	NCD	HFD	5%BB in HFD	10%BB in HFD
% of Lymphocytes					
NK	CD3-NK1.1+	3.9±0.3	3.3±0.4	3.6±0.5	2.6±0.2
T cells	CD3+	22.4±2.1	25.2±2.8	24.3±1.8	29.3±1.8
% of all T cells					
T helper (Th)	CD3+CD4+	49.7±3.3	50.0±2.5	50.3±1.8	50.7±2.5
Cytotoxic T (Tc)	CD3+CD8+	14.9±1.0	18.2±1.3	17.8±1.5	14.6±2.2
**NKT**	CD3+CD8+NK1.1+	16.0±2.1	10.1±1.3	12.5±1.6	5.1±0.5^a^
**Mature NKT**	CD3+CD8+NK1.1+^high^	1.8±0.3	1.6±0.3	1.5±0.3	0.2±0.0^a^
**IFNg producing**	CD3+IFNg+	20.2±1.6	20.3±1.8	20.3±1.7	12.1±1.8^a^
IFNg producing Tc cells	CD3+CD8+ IFNg+	6.9±0.7	8.2±0.6	7.8±0.8	5.2±1.3
% of T helper cells					
**Th 1**	CD3+CD4+IFNg+IL4-	15.6±1.5	13.6±1.1	13.0±11	7.2±0.7^a^
Th 2	CD3+CD4+IFNg-IL4+	0.5±0.1	0.4±0.1	0.7±0.3	0.6±0.1
Th 17	CD3+CD4+IFNg-IL17+	0.6±0.2	0.6±0.3	0.5±0.1	0.3±0.1
**Th1/Th2**		37±6	40±5	27±7	13±2^a^

NK – Natural killer cells, NKT – Natural killer T cells.

a) Significantly different as compared to HFD (p<0.05, Mann Whitney U-Test with Bonferroni's correction).

The splenic lymphocytes; T-cells and Natural Killer Cells (NK) and sub-populations of T-cells in mice fed with normal control diet (NCD), high-fat diet (HFD) and 5% or 10% BB in HFD for 12–14 weeks. Data from the FACS analyses is given as percentage of the respective cell population and represents the mean values of n = 6–11 mice per diet group ± SEM.

The relative numbers of T cells, NK cells and NKT cells and specific subtypes of T cells correlated with the serum levels of some pro- and anti-inflammatory cytokines (Table S6 in S1 Panel, supporting phenotypic characteristics). Perhaps most notably the relative numbers of Th1 cells correlated positively with several cytokines associated with the type 1 cell-mediated pro-inflammatory responses (MIP-1, TNF-α, IL-5 and IL-7), as would be expected.

### Effects on blood pressure

**Figure 4:**
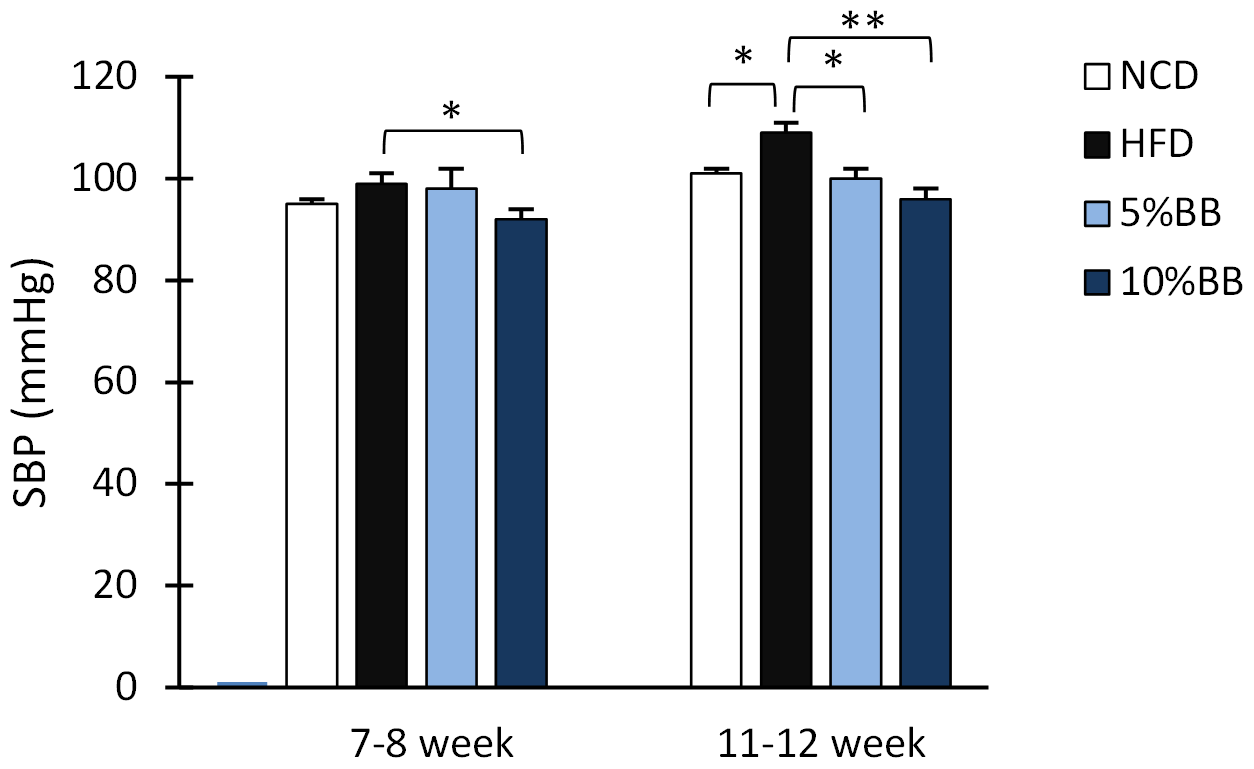
Effects of feeding NCD, HFD, or bilberries in HFD on the systolic blood pressure in mice. Systolic blood pressure (SBP) was measured by the non-invasive Tail Cuffs photoelectric method at 7–8 and 11–12 weeks after the start of the diets. The bars represent the means and SEM of n = 10–14 mice per diet group (see details in Table S5 in S1 Panel, supporting phenotypic characteristics). Asterisk (*) indicates the significant differences of HFD group from NCD and 5% BB and 10% BB in HFD groups (*p<0.05, **p<0.001, Mann Whitney U-Test with Bonferroni's correction).

The systolic blood pressure (SBP) in mice receiving HFD was moderately increased at 7–8 weeks (+4 mmHg) and significantly increased at 11–12 weeks (+8 mmHg) (Figure 4). In contrast, mice fed with BB in HFD did not develop hypertension. This difference was observed as mice in 10% BB in HFD had significantly lower SBP (−6 mmHg) than mice fed HFD alone, as early as 7–8 weeks after starting the diets (p<0.05). Moreover, both BB fed groups exhibited significantly lower blood pressures than mice fed solely a HFD at 11–12 weeks (Figure 4). No significant differences were observed in heart rates (Table S5 in S1 Panel, supporting phenotypic characteristics).

**Figure 5:**
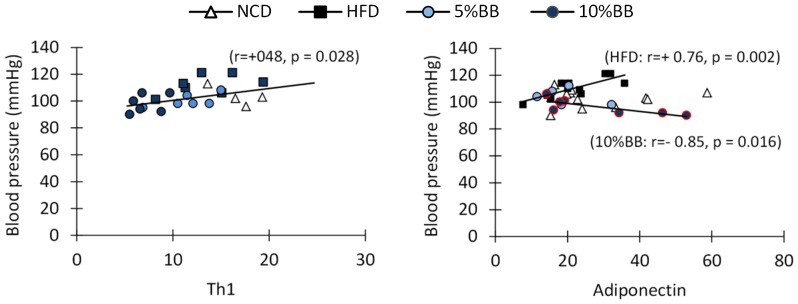
Correlations of T helper type 1 cells (Th 1) and serum adiponectin with blood pressure in mice fed NCD, HFD or bilberries in HFD. Correlations of Th1 and serum adiponectin (µg/mL) with blood pressure in mice were analyzed after feeding diets for 12–14 weeks. The relative number of splenic Th 1 cells (CD3+CD4+IFNg+IL4-) of all T cells (CD3+) was used in calculating the correlation. The values of the group fed 10% BB in HFD are highlighted by red circles. Each symbol represents an individual measurement. The lines indicate significant correlations (Spearman's test).

The blood pressure was found to correlate with the intake of sodium and levels of pro-inflammatory cytokine IFN-γ and the relative amount of cellular markers of inflammation (Figure S5 and Table S6 in S1 Panel, supporting phenotypic characteristics). The relative amount of several subtypes of T cells correlated positively with blood pressure: i.e. the amounts of Th1 (r = 0.47, p = 0.025), mature NKT cells (r = 0.52, p = 0.011), INF-γ producing cells (r = 0.57, p = 0.004) and Tc cells producing INF-γ (r = 0.60, p = 0.002) were higher in elevated blood pressure (Figure 5 and Figure S5 in S1 Panel, supporting phenotypic characteristics). In addition, blood pressure correlated negatively with the adiponectin level in mice fed with BB and positively in mice fed HFD alone (Figure 5). The adiponectin level correlated also positively with weight for mice fed HFD (0.56, p = 0.038) but not for mice fed BB.

## Discussion

The present study aimed to test whether whole bilberries (freeze dried) as a part of the high-fat diet (HFD) can prevent development of obesity and related metabolic and immunological disturbances. In an attempt to assess this problem we used the HFD mouse model [14]–[17], [44] and we measured several metabolic and immunological characteristics known to be associated with obesity and its adverse health effects. The results showed that bilberries effectively improve the inflammatory status and reduce hypertension caused by HFD, while having only minor effects on other obesity associated metabolic disturbances.

### HFD associated weight gain and disturbances in glucose metabolism

The HFD mouse model is distinctive in increasing weight gain and producing hypertension, hyperlipidaemia, and altering several markers of inflammatory status as shown here and in previous studies [5], [14]–[16], [38]. Weight gain induced by HFD was significantly reduced by 10% BB added to HFD, and also the body fat % tended to be reduced in parallel with the change in adipocyte secreted leptin. These results are in agreement with those of Tsuda et al. 2003 [45] and Heyman et al. 2014 [46] who showed that high intake of ANCs can reduce the HFD induced weight gain in mouse models of obesity or diabetes. ANCs supplied in drinking water or in extracts are better absorbed [47], and have also been found to decrease adiposity and improve insulin sensitivity [26], [29]. As expected, mice fed HFD also became glucose intolerant and insulin resistant. However, we were unable to observe changes in relative insulin sensitivity. One reason for this phenomenon may be that the strain C57BL/6J is known to be more resistant than other mouse strains to HFD produced changes in insulin sensitivity [15]. Previous studies have found that bilberry extracts can increase insulin sensitivity at 5–8 weeks in male KK-A^y^ mice model of T2D [27] and 4% (w/w) blueberries at 8 weeks for the HFD mouse model [6]. The short fasting time (2 h) as well as lower insulin dose may explain the differences between our findings and those by others using mice fasted for 6 or 14 h [6], [27]. The fasting time is a crucial factor affecting insulin receptor signalling pathways [48]. Thus these results indicate that while whole bilberries are effective in increasing insulin sensitivity at the early stages of obesity, they are unable to counteract the disturbance produced over a longer period of HFD feeding.

### Effects of bilberries on HFD associated inflammation

HFD is known to cause low-grade chronic inflammation that is characterized by the interplay of several cell types and mediators, cytokines and adipokines [5]. In this study, HFD tended to increase pro-inflammatory cytokines in parallel with weight gain and increased body fat. However, the levels of several parameters of inflammation were reduced by addition of bilberries to HFD, approaching the levels found for NCD fed mice. Bilberry intake appeared to consistently direct the HFD induced immune profile away from the type 1 cell-mediated immune responses also linked with the obesity associated cardiometabolic complications and hypertension [5], [49]–[51]. Namely, BB tended to decrease the levels of IL-1β, IL-2, IL-6, TNF-α, MCP-1 and collectively Th1 cytokines as well as the number of Th1 cells. Similarily, in a Zucker rat model of MetS 8% (w/w) blueberries (*Vaccinium angustifolium*) were found to decrease the levels of pro-inflammatory cytokines (IL-6, TNF-α and CRP) and to increase the adiponectin levels [9]. These results are in line with human trials conducted by our group [8] and others [7]. Karlsen et al. (2010) reported the reduction in the levels of CRP, IL-6, and IL-15 by bilberries in the diet [7].

In particular, IL-15 was nearly tripled by the HFD in the present study and not detected in either of the groups receiving BB added to HFD. The reduction of NKT cells for BB fed mice is in line with this since IL-15 is known to regulate the activation and proliferation of T, NK and NKT cells [39]. This is particularly interesting since the NKT cells are proposed to play a crucial role as the initiators of obesity associated inflammation and exacerbate impaired glucose homeostasis [52]. Also the level of IFN-γ was increased for HFD fed animals and reduced to non-detectable levels by bilberries. These findings were supported by the cellular analyses as the number of INF-γ producing cells were reduced for mice fed with the high dose of BB. INF-γ has previously been shown to mediate obesity associated inflammation in adipose tissue. This may be partially mediated by its up-regulating effect on the expression of the gene encoding for MCP-1 [53], which is known to have a pivotal role in the recruitment and polarization of monocytes to the sites of inflammation in obesity [54]. Notably, and in alignment to this, the serum levels of MCP-1 were reduced by bilberries in the present study. This supports our previous findings displaying reduced expression of MCP-1 receptor gene (*CCR-2*) in human monocytes after feeding a bilberry enriched diet [8]. The decreases in the serum cytokine levels observed may also reflect changes in adipocyte gene expression, as 4% whole blueberry powder (*Vaccinium ashei and V. corymbosum*) has been shown to decrease *IL-6, MCP-1* and *TNF-α* expression in the HFD mouse model [6].

Resistin, an adipokine that has been linked to the onset of obesity-associated diabetes [55], was reduced in BB enriched diets. Anthocyanin rich juice has been shown to reduce resistin levels [56] and our results support this finding. The regulation of resistin levels could be a target for anti-diabetic therapy, especially through its association with the inflammatory process [57]. Overall, resistin has pro-inflammatory properties and can regulate *MCP-1* expression [58]. Further, it has also been found to exacerbate inflammatory processes in chronic endotoxemia [59], which could be reduced by bilberries in rats and humans [8], [60]. In addition, the levels of G-CSF were increased by BB added to the diet. The relevance of this finding is unclear, but interestingly it has been linked with enhanced regeneration of cardiovascular tissues and thus could have beneficial cardiovascular effects [61].

### Effects of bilberries on HFD associated hypertension

Hypertension is one of the the criteria for diagnosing MetS and commonly associated with obesity. In this study we demonstrated that bilberries can prevent the development of hypertension in a dose dependent manner. The pathophysiological mechanism of hypertension is multifactorial and includes oxidative stress, inflammation, the renin-angiotensin system and autoimmune vascular dysfunction [62]–[64]. Hypertension may be reduced by food constituents, some of which may act like anti-hypertensive medication e.g. as angiotensin converting enzyme (ACE) inhibitors, angiotensin receptor blockers, β-blockers, Ca-channel blockers, central α-agonists, direct renin inhibitors, direct vasodilators or diuretics [65]. Our findings are in line with previous reports [31], [66]. Spontaneously hypertensive rats have been reported to have marked reduction in blood pressure after supplementation with 3% (w/w) freeze-dried blueberries (*Vaccinium angustifolium*) [66]. The authors postulated that this reduction was due to reduced oxidative stress in the kidneys. Furthermore, Elks and colleagues showed that improved renal catalase and glutathione activities were likely behind the reduced blood pressure using 2% (w/w) blueberries supplemented diet *(Vaccinium spp.)* [31]. Similar findings in the reduction of BP have also been demonstrated with other sources of ANCs [30]. Nevertheless, our study reveals for the first time that whole *Vaccinium myrtillus* berries clearly prevent the development of chronic hypertension. The mechanisms underlying these effects need still further investigation.

Our results further suggest that prevention of chronic hypertension by bilberries may be mediated by anti-inflammatory action. In HFD fed mice, the SBP correlated with a number of inflammatory markers and sodium intake. The blood pressure correlated with pro-inflammatory cells and cytokines, especially Th1 cells and their signature cytokine INF- γ. Similarly, increased levels of IL-6, leptin, and resistin have been previously linked to increased blood pressure and increased level of adiponectin to reduced blood pressure [64], [67]. In our study adiponectin was linked to reduced blood pressure for BB fed mice, but surprisingly an opposite correlation was found in animals fed HFD alone. This may be due to an increase in the amount of adiponectin secreting adipose tissue in mice with HFD, which is supported by the observed direct association between adiponectin and weight. The increased association may also be due to the ability of adiponectin to induce Th1 differentiation [68], which is affected by bilberries. Furthermore, there is also evidence from studies with mice [69] and humans [70], that changes in the ratios of adiponectin isoforms could affect hypertension and inflammation in MetS.

Long-term effects on hypertension may be attributed to the reduced weight gain, oxidative stress and inflammation, whereas a short-term (acute) effects are most likely due to altered enzyme activities or compounds influencing vasodilation directly, as has been found with grape polyphenols [71] and ANC extracts from bilberries [72]. Since ANCs have been shown to have acute vascular effects in rodent models [73], [74] and in humans [75], the relaxation of arterial walls by ACNs is a possible mechanism for an acute decrease in blood pressure by bilberries that may in part play a role in the observed long term reduction in blood pressure. Blueberry intake has been associated with a reduction of blood pressure also in humans [76], but findings in intervention studies have thus far been inconsistent [8], [77], [78].

### Strengths and limits of the study

The strength of the present study is in combining the major phenotypic features of the metabolic syndrome with the analysis of several immunological markers. Some limitations still have to be considered before arriving at final conclusions for this study. We fed the mice with HFD and bilberries for three months and explored the effects of berries on development of obesity-associated symptoms. However, the duration of our study was relatively short, covering only the juvenile-early adult age of the animals. Fluctuations in feeding behaviour over a longer period can occur and influence the results as we observed. Moreover, a rather short fasting period was used in our study to better reflect the non-stressed physiological state. This may have introduced some variation in the parameters measured. Nevertheless, logical trends concerning the effects of bilberries were found both with individual and combined parameters of inflammatory cells and cytokines. Furthermore, studies utilizing gene expression patterns, metabolomics and microbiomics in target tissues and organs of the obesity-associated effects may further unravel more detailed mechanisms underlying these health effects.

## Conclusions

We have shown here with the HFD mouse model that bilberries can ameliorate or prevent metabolic disturbances associated with developing obesity, especially systemic low-grade inflammation and hypertension. The effect of whole bilberries on hypertension associated with HFD consumption has not been examined in detail prior to this study. Since the obesity associated pathologies are usually clustered (IR-T2D-hypertension) [79], dietary approaches influencing all of these pathologies may be more beneficial than treating hypertension or abnormal glucose levels alone. Our findings attest to the epidemiological [76], [80], [81] and clinical [82] evidence supporting bilberries as a promising candidate for the prevention of obesity related hypertension and low-grade inflammation.

## Supporting Information

S1 Panel **Supporting phenotypic characteristics.** Figures (S1–S5) and tables (S3–S6) on the markers of glucose and lipid metabolism, blood pressure and inflammation in mice fed NCD, HFD or HFD with bilberries: Figure S1, Insulin sensitivity at 7 weeks in mice fed NCD, HFD or HFD with bilberries. Figure S2, Insulin sensitivity at 11–12 weeks in mice fed NCD, HFD or HFD with bilberries. Figure S3, Glucose tolerance in mice fed NCD, HFD or HFD with bilberries. Figure S4, The net-effect of serum pro-inflammatory cytokines in mice fed NCD, HFD or HFD with bilberries. Figure S5, Correlation of inflammatory cells and adiponectin with blood pressure. Table S3, Blood lipids of mice fed NCD, HFD or HFD with bilberries. Table S4, Serum levels of pro- and anti-inflammatory cytokines in mice fed NCD, HFD or HFD with bilberries. Table S5, Systolic blood pressure and heart rate in mice NCD, HFD or HFD with bilberries. Table S6, Correlation of inflammatory cells with cytokines and adipokines.
(PDF)

S1 Table **Details of the diets.** Contents of the diets as per weight and energy, profiles of anthocyanins, lipids and salt.
(PDF)

S2 Table **Intakes of food, water and energy.** Mean intakes of food and water consumed per animal, calculated values of energy and diet per animal weight at the beginning, middle and the end of the study.
(PDF)

## References

[1] Ouchi N, Parker JL, Lugus JJ, Walsh K (2011) Adipokines in inflammation and metabolic disease. Nat Rev Immunol 11:85–97. 21252989 10.1038/nri2921 PMC3518031

[2] Colosia AD, Palencia R, Khan S (2013) Prevalence of hypertension and obesity in patients with type 2 diabetes mellitus in observational studies: A systematic literature review. Diabetes Metab Syndr Obes 6:327–338. 24082791 10.2147/DMSO.S51325 PMC3785394

[3] Xu H, Barnes GT, Yang Q, Tan G, Yang D, et al (2003) Chronic inflammation in fat plays a crucial role in the development of obesity-related insulin resistance. J Clin Invest 112:1821–1830. 14679177 10.1172/JCI19451 PMC296998

[4] Winer S, Winer DA (2012) The adaptive immune system as a fundamental regulator of adipose tissue inflammation and insulin resistance. Immunol Cell Biol 90:755–762. 22231651 10.1038/icb.2011.110

[5] Xu H (2013) Obesity and metabolic inflammation. Drug Discov Today Dis Mech 10:21–25. 24003334 10.1016/j.ddmec.2013.03.006 PMC3758492

[6] DeFuria J, Bennett G, Strissel KJ, Perfield JW 2nd, Milbury PE, et al (2009) Dietary blueberry attenuates whole-body insulin resistance in high fat-fed mice by reducing adipocyte death and its inflammatory sequelae. J Nutr 139:1510–1516. 19515743 10.3945/jn.109.105155 PMC2709302

[7] Karlsen A, Paur I, Bohn SK, Sakhi AK, Borge GI, et al (2010) Bilberry juice modulates plasma concentration of NF-kappaB related inflammatory markers in subjects at increased risk of CVD. Eur J Nutr 49:345–355. 20119859 10.1007/s00394-010-0092-0

[8] Kolehmainen M, Mykkanen O, Kirjavainen PV, Leppanen T, Moilanen E, et al (2012) Bilberries reduce low-grade inflammation in individuals with features of metabolic syndrome. Mol Nutr Food Res 56:1501–1510. 22961907 10.1002/mnfr.201200195

[9] Vendrame S, Daugherty A, Kristo AS, Riso P, Klimis-Zacas D (2013) Wild blueberry (vaccinium angustifolium) consumption improves inflammatory status in the obese zucker rat model of the metabolic syndrome. J Nutr Biochem 24:1508–1512. 23465589 10.1016/j.jnutbio.2012.12.010

[10] Kolb H, Mandrup-Poulsen T (2010) The global diabetes epidemic as a consequence of lifestyle-induced low-grade inflammation. Diabetologia 53:10–20. 19890624 10.1007/s00125-009-1573-7

[11] Nishimura S, Manabe I, Nagasaki M, Eto K, Yamashita H, et al (2009) CD8+ effector T cells contribute to macrophage recruitment and adipose tissue inflammation in obesity. Nat Med 15:914–920. 19633658 10.1038/nm.1964

[12] Ohmura K, Ishimori N, Ohmura Y, Tokuhara S, Nozawa A, et al (2010) Natural killer T cells are involved in adipose tissues inflammation and glucose intolerance in diet-induced obese mice. Arterioscler Thromb Vasc Biol 30:193–199. 19910631 10.1161/ATVBAHA.109.198614

[13] Strissel KJ, DeFuria J, Shaul ME, Bennett G, Greenberg AS, et al (2010) T-cell recruitment and Th1 polarization in adipose tissue during diet-induced obesity in C57BL/6 mice. Obesity (Silver Spring) 18:1918–1925. 20111012 10.1038/oby.2010.1 PMC2894258

[14] Mills E, Kuhn CM, Feinglos MN, Surwit R (1993) Hypertension in CB57BL/6J mouse model of non-insulin-dependent diabetes mellitus. Am J Physiol 264:R73–8. 8430889 10.1152/ajpregu.1993.264.1.R73

[15] Rossmeisl M, Rim JS, Koza RA, Kozak LP (2003) Variation in type 2 diabetes—related traits in mouse strains susceptible to diet-induced obesity. Diabetes 52:1958–1966. 12882911 10.2337/diabetes.52.8.1958

[16] Collins S, Martin TL, Surwit RS, Robidoux J (2004) Genetic vulnerability to diet-induced obesity in the C57BL/6J mouse: Physiological and molecular characteristics. Physiol Behav 81:243–248. 15159170 10.1016/j.physbeh.2004.02.006

[17] Montgomery MK, Hallahan NL, Brown SH, Liu M, Mitchell TW, et al (2013) Mouse strain-dependent variation in obesity and glucose homeostasis in response to high-fat feeding. Diabetologia 56:1129–1139. 23423668 10.1007/s00125-013-2846-8

[18] Riihinen K, Jaakola L, Kärenlampi S, Hohtola A (2008) Organ-specific distribution of phenolic compounds in bilberry (vaccinium myrtillus) and ‘northblue’ blueberry (vaccinium corymbosum x V. angustifolium). Food Chem 110:156–160. 26050178 10.1016/j.foodchem.2008.01.057

[19] Koponen JM, Happonen AM, Mattila PH, Torronen AR (2007) Contents of anthocyanins and ellagitannins in selected foods consumed in finland. J Agric Food Chem 55:1612–1619. 17261015 10.1021/jf062897a

[20] Ovaskainen ML, Torronen R, Koponen JM, Sinkko H, Hellstrom J, et al (2008) Dietary intake and major food sources of polyphenols in finnish adults. J Nutr 138:562–566. 18287367 10.1093/jn/138.3.562

[21] He J, Giusti MM (2010) Anthocyanins: Natural colorants with health-promoting properties. Annu Rev Food Sci Technol 1:163–187. 22129334 10.1146/annurev.food.080708.100754

[22] Nile SH, Park SW (2014) Edible berries: Bioactive components and their effect on human health. Nutrition 30:134–144. 24012283 10.1016/j.nut.2013.04.007

[23] Ghosh D, Konishi T (2007) Anthocyanins and anthocyanin-rich extracts: Role in diabetes and eye function. Asia Pac J Clin Nutr 16:200–208. 17468073

[24] Helmstadter A, Schuster N (2010) Vaccinium myrtillus as an antidiabetic medicinal plant—research through the ages. Pharmazie 65:315–321. 20503920

[25] Jayaprakasam B, Olson LK, Schutzki RE, Tai MH, Nair MG (2006) Amelioration of obesity and glucose intolerance in high-fat-fed C57BL/6 mice by anthocyanins and ursolic acid in cornelian cherry (cornus mas). J Agric Food Chem 54:243–248. 16390206 10.1021/jf0520342

[26] Prior RL, Wilkes SE, Rogers TR, Khanal RC, Wu X, et al (2010) Purified blueberry anthocyanins and blueberry juice alter development of obesity in mice fed an obesogenic high-fat diet. J Agric Food Chem 58:3970–3976. 20148514 10.1021/jf902852d

[27] Takikawa M, Inoue S, Horio F, Tsuda T (2010) Dietary anthocyanin-rich bilberry extract ameliorates hyperglycemia and insulin sensitivity via activation of AMP-activated protein kinase in diabetic mice. J Nutr 140:527–533. 20089785 10.3945/jn.109.118216

[28] Prior RL, Wu X, Gu L, Hager TJ, Hager A, et al (2008) Whole berries versus berry anthocyanins: Interactions with dietary fat levels in the C57BL/6J mouse model of obesity. J Agric Food Chem 56:647–653. 18211017 10.1021/jf071993o

[29] Prior RL, Wu X, Gu L, Hager T, Hager A, et al (2009) Purified berry anthocyanins but not whole berries normalize lipid parameters in mice fed an obesogenic high fat diet. Mol Nutr Food Res 53:1406–1418. 19743407 10.1002/mnfr.200900026

[30] Shindo M, Kasai T, Abe A, Kondo Y (2007) Effects of dietary administration of plant-derived anthocyanin-rich colors to spontaneously hypertensive rats. J Nutr Sci Vitaminol (Tokyo) 53:90–93. 17484387 10.3177/jnsv.53.90

[31] Elks CM, Reed SD, Mariappan N, Shukitt-Hale B, Joseph JA, et al (2011) A blueberry-enriched diet attenuates nephropathy in a rat model of hypertension via reduction in oxidative stress. PLoS One 6:e24028. 21949690 10.1371/journal.pone.0024028 PMC3174132

[32] Rodriguez-Mateos A, Ishisaka A, Mawatari K, Vidal-Diez A, Spencer JP, et al (2013) Blueberry intervention improves vascular reactivity and lowers blood pressure in high-fat-, high-cholesterol-fed rats. Br J Nutr 109:1746–1754. 23046999 10.1017/S0007114512003911

[33] Mykkanen OT, Kalesnykas G, Adriaens M, Evelo CT, Torronen R, et al (2012) Bilberries potentially alleviate stress-related retinal gene expression induced by a high-fat diet in mice. Mol Vis 18:2338–2351. 22993483 PMC3444297

[34] Tauriainen E, Luostarinen M, Martonen E, Finckenberg P, Kovalainen M, et al (2011) Distinct effects of calorie restriction and resveratrol on diet-induced obesity and fatty liver formation. J Nutr Metab 2011:525094. 21977315 10.1155/2011/525094 PMC3184417

[35] Sharma S, Fulton S (2013) Diet-induced obesity promotes depressive-like behaviour that is associated with neural adaptations in brain reward circuitry. Int J Obes (Lond) 37:382–389. 22508336 10.1038/ijo.2012.48

[36] Thomas EL, Saeed N, Hajnal JV, Brynes A, Goldstone AP, et al (1998) Magnetic resonance imaging of total body fat. J Appl Physiol (1985) 85:1778–1785. 9804581 10.1152/jappl.1998.85.5.1778

[37] Heikkinen S, Argmann CA, Champy MF, Auwerx J (2007) Evaluation of glucose homeostasis. Curr Protoc Mol Biol Chapter 29: Unit 29B.3. 10.1002/0471142727.mb29b03s77 18265403

[38] Kleemann R, Zadelaar S, Kooistra T (2008) Cytokines and atherosclerosis: A comprehensive review of studies in mice. Cardiovasc Res 79:360–376. 18487233 10.1093/cvr/cvn120 PMC2492729

[39] Akdis M, Burgler S, Crameri R, Eiwegger T, Fujita H, et al (2011) Interleukins, from 1 to 37, and interferon-gamma: Receptors, functions, and roles in diseases. J Allergy Clin Immunol 127:701–21.e1-70. 21377040 10.1016/j.jaci.2010.11.050

[40] Seruga B, Zhang H, Bernstein LJ, Tannock IF (2008) Cytokines and their relationship to the symptoms and outcome of cancer. Nat Rev Cancer 8:887–899. 18846100 10.1038/nrc2507

[41] Kirjavainen PV, ElNezami HS, Salminen SJ, Ahokas JT, Wright PF (1999) Effects of orally administered viable lactobacillus rhamnosus GG and propionibacterium freudenreichii subsp. shermanii JS on mouse lymphocyte proliferation. Clin Diagn Lab Immunol 6:799–802. 10548566 10.1128/cdli.6.6.799-802.1999 PMC95778

[42] Kirjavainen PV, Pautler S, Baroja ML, Anukam K, Crowley K, et al (2009) Abnormal immunological profile and vaginal microbiota in women prone to urinary tract infections. Clin Vaccine Immunol 16:29–36. 19020112 10.1128/CVI.00323-08 PMC2620669

[43] Johns C, Gavras I, Handy DE, Salomao A, Gavras H (1996) Models of experimental hypertension in mice. Hypertension 28:1064–1069. 8952597 10.1161/01.hyp.28.6.1064

[44] Surwit RS, Kuhn CM, Cochrane C, McCubbin JA, Feinglos MN (1988) Diet-induced type II diabetes in C57BL/6J mice. Diabetes 37:1163–1167. 3044882 10.2337/diab.37.9.1163

[45] Tsuda T, Horio F, Uchida K, Aoki H, Osawa T (2003) Dietary cyanidin 3-O-beta-D-glucoside-rich purple corn color prevents obesity and ameliorates hyperglycemia in mice. J Nutr 133:2125–2130. 12840166 10.1093/jn/133.7.2125

[46] Heyman L, Axling U, Blanco N, Sterner O, Holm C, et al (2014) Evaluation of beneficial metabolic effects of berries in high-fat fed C57BL/6J mice. Journal of Nutrition and Metabolism 2014:12. 10.1155/2014/403041 PMC3941780 24669315

[47] Yang M, Koo SI, Song WO, Chun OK (2011) Food matrix affecting anthocyanin bioavailability: Review. Curr Med Chem 18:291–300. 21110799 10.2174/092986711794088380

[48] Agouni A, Owen C, Czopek A, Mody N, Delibegovic M (2010) In vivo differential effects of fasting, re-feeding, insulin and insulin stimulation time course on insulin signaling pathway components in peripheral tissues. Biochem Biophys Res Commun 401:104–111. 20833131 10.1016/j.bbrc.2010.09.018

[49] Mathieu P, Lemieux I, Despres JP (2010) Obesity, inflammation, and cardiovascular risk. Clin Pharmacol Ther 87:407–416. 20200516 10.1038/clpt.2009.311

[50] De Ciuceis C, Rossini C, La Boria E, Porteri E, Petroboni B, et al**.** (2014) Immune mechanisms in hypertension. High Blood Press Cardiovasc Prev. 10.1007/s40292-014-0040-9 24446309

[51] Schiffrin EL (2014) Immune mechanisms in hypertension and vascular injury. Clin Sci (Lond) 126:267–274. 24144355 10.1042/CS20130407

[52] Wu Y, Wu T, Wu J, Zhao L, Li Q, et al (2013) Chronic inflammation exacerbates glucose metabolism disorders in C57BL/6J mice fed with high-fat diet. J Endocrinol 219:195–204. 24029730 10.1530/JOE-13-0160

[53] Rocha VZ, Folco EJ, Sukhova G, Shimizu K, Gotsman I, et al (2008) Interferon-gamma, a Th1 cytokine, regulates fat inflammation: A role for adaptive immunity in obesity. Circ Res 103:467–476. 18658050 10.1161/CIRCRESAHA.108.177105 PMC2740384

[54] Kanda H, Tateya S, Tamori Y, Kotani K, Hiasa K, et al (2006) MCP-1 contributes to macrophage infiltration into adipose tissue, insulin resistance, and hepatic steatosis in obesity. J Clin Invest 116:1494–1505. 16691291 10.1172/JCI26498 PMC1459069

[55] Steppan CM, Bailey ST, Bhat S, Brown EJ, Banerjee RR, et al (2001) The hormone resistin links obesity to diabetes. Nature 409:307–312. 11201732 10.1038/35053000

[56] Graf D, Seifert S, Jaudszus A, Bub A, Watzl B (2013) Anthocyanin-rich juice lowers serum cholesterol, leptin, and resistin and improves plasma fatty acid composition in fischer rats. PLoS One 8:e66690. 23825152 10.1371/journal.pone.0066690 PMC3688949

[57] Pang SS, Le YY (2006) Role of resistin in inflammation and inflammation-related diseases. Cell Mol Immunol 3:29–34. 16549046

[58] Park HK, Ahima RS (2013) Resistin in rodents and humans. Diabetes Metab J 37:404–414. 24404511 10.4093/dmj.2013.37.6.404 PMC3881324

[59] Park HK, Qatanani M, Briggs ER, Ahima RS, Lazar MA (2011) Inflammatory induction of human resistin causes insulin resistance in endotoxemic mice. Diabetes 60:775–783. 21282361 10.2337/db10-1416 PMC3046838

[60] Sautebin L, Rossi A, Serraino I, Dugo P, Di Paola R, et al (2004) Effect of anthocyanins contained in a blackberry extract on the circulatory failure and multiple organ dysfunction caused by endotoxin in the rat. Planta Med 70:745–752. 15368665 10.1055/s-2004-827206

[61] Takano H, Ueda K, Hasegawa H, Komuro I (2007) G-CSF therapy for acute myocardial infarction. Trends Pharmacol Sci 28:512–517. 17888521 10.1016/j.tips.2007.09.002

[62] Stas SN, El-Atat FA, Sowers JR (2004) Pathogenesis of hypertension in diabetes. Rev Endocr Metab Disord 5:221–225. 15211093 10.1023/B:REMD.0000032410.75638.da

[63] Harrison DG, Guzik TJ, Goronzy J, Weyand C (2008) Is hypertension an immunologic disease? Curr Cardiol Rep 10:464–469. 18950555 10.1007/s11886-008-0073-6

[64] Vlasova M, Purhonen AK, Jarvelin MR, Rodilla E, Pascual J, et al (2010) Role of adipokines in obesity-associated hypertension. Acta Physiol (Oxf) 200:107–127. 20653609 10.1111/j.1748-1716.2010.02171.x

[65] Houston M (2013) Nutrition and nutraceutical supplements for the treatment of hypertension: Part I. J Clin Hypertens (Greenwich). 15:752–757. 10.1111/jch.12188 PMC8033896 24088285

[66] Shaughnessy KS, Boswall IA, Scanlan AP, Gottschall-Pass KT, Sweeney MI (2009) Diets containing blueberry extract lower blood pressure in spontaneously hypertensive stroke-prone rats. Nutr Res 29:130–138. 19285604 10.1016/j.nutres.2009.01.001

[67] Papadopoulos DP, Makris TK, Krespi PG, Poulakou M, Stavroulakis G, et al (2005) Adiponectin and resistin plasma levels in healthy individuals with prehypertension. J Clin Hypertens (Greenwich) 7:729–733. 16330895 10.1111/j.1524-6175.2005.04888.x PMC8109703

[68] Cheng X, Folco EJ, Shimizu K, Libby P (2012) Adiponectin induces pro-inflammatory programs in human macrophages and CD4+ T cells. J Biol Chem 287:36896–36904. 22948153 10.1074/jbc.M112.409516 PMC3481292

[69] Aoqui C, Chmielewski S, Scherer E, Eissler R, Sollinger D, et al (2014) Microvascular dysfunction in the course of metabolic syndrome induced by high-fat diet. Cardiovasc Diabetol 13:31–2840–13-31. 24490784 10.1186/1475-2840-13-31 PMC3916304

[70] Baumann M, von Eynatten M, Dan L, Richart T, Kouznetsova T, et al (2009) Altered molecular weight forms of adiponectin in hypertension. J Clin Hypertens (Greenwich) 11:11–16. 19125853 10.1111/j.1751-7176.2008.00057.x PMC8673079

[71] Mendes A, Desgranges C, Cheze C, Vercauteren J, Freslon JL (2003) Vasorelaxant effects of grape polyphenols in rat isolated aorta. possible involvement of a purinergic pathway. Fundam Clin Pharmacol 17:673–681. 15015712 10.1046/j.1472-8206.2003.00198.x

[72] Bell DR, Gochenaur K (2006) Direct vasoactive and vasoprotective properties of anthocyanin-rich extracts. J Appl Physiol (1985) 100:1164–1170. 16339348 10.1152/japplphysiol.00626.2005

[73] Kalea AZ, Clark K, Schuschke DA, Kristo AS, Klimis-Zacas DJ (2010) Dietary enrichment with wild blueberries (vaccinium angustifolium) affects the vascular reactivity in the aorta of young spontaneously hypertensive rats. J Nutr Biochem 21:14–22. 19157824 10.1016/j.jnutbio.2008.09.005

[74] Horrigan LA, Holohan CA, Lawless GA, Murtagh MA, Williams CT, et al (2013) Blueberry juice causes potent relaxation of rat aortic rings via the activation of potassium channels and the H(2)S pathway. Food Funct 4:392–400. 23175156 10.1039/c2fo30205e

[75] Jennings A, Welch AA, Fairweather-Tait SJ, Kay C, Minihane AM, et al (2012) Higher anthocyanin intake is associated with lower arterial stiffness and central blood pressure in women. Am J Clin Nutr 96:781–788. 22914551 10.3945/ajcn.112.042036

[76] Cassidy A, O'Reilly EJ, Kay C, Sampson L, Franz M, et al (2011) Habitual intake of flavonoid subclasses and incident hypertension in adults. Am J Clin Nutr 93:338–347. 21106916 10.3945/ajcn.110.006783 PMC3021426

[77] Basu A, Du M, Leyva MJ, Sanchez K, Betts NM, et al (2010) Blueberries decrease cardiovascular risk factors in obese men and women with metabolic syndrome. J Nutr 140:1582–1587. 20660279 10.3945/jn.110.124701 PMC2924596

[78] Riso P, Klimis-Zacas D, Del Bo' C, Martini D, Campolo J, et al (2013) Effect of a wild blueberry (vaccinium angustifolium) drink intervention on markers of oxidative stress, inflammation and endothelial function in humans with cardiovascular risk factors. Eur J Nutr 52:949–961. 22733001 10.1007/s00394-012-0402-9

[79] Reaven GM (2011) Relationships among insulin resistance, type 2 diabetes, essential hypertension, and cardiovascular disease: Similarities and differences. J Clin Hypertens (Greenwich) 13:238–243. 21466618 10.1111/j.1751-7176.2011.00439.x PMC8673405

[80] Wedick NM, Pan A, Cassidy A, Rimm EB, Sampson L, et al (2012) Dietary flavonoid intakes and risk of type 2 diabetes in US men and women. Am J Clin Nutr 95:925–933. 22357723 10.3945/ajcn.111.028894 PMC3302366

[81] Muraki I, Imamura F, Manson JE, Hu FB, Willett WC, et al (2013) Fruit consumption and risk of type 2 diabetes: Results from three prospective longitudinal cohort studies. BMJ 347:f5001. 23990623 10.1136/bmj.f5001 PMC3978819

[82] Joseph SV, Edirisinghe I, Burton-Freeman BM (2014) Berries: Anti-inflammatory effects in humans. J Agric Food Chem. 10.1021/jf4044056 24512603

