# Supplementary material for: Wild Blueberries (Vaccinium myrtillus) Alleviate Inflammation and Hypertension Associated with Developing Obesity in Mice Fed with a High-Fat Diet
Source: PLoS One. 2014 Dec 12;9(12):e114790. doi: 10.1371/journal.pone.0114790 (PMC4264776; doi:10.1371/journal.pone.0114790)
Supplement: S1 Panel — Supporting phenotypic characteristics. Figures (S1–S5) and tables (S3–S6) on the markers of glucose and lipid metabolism, blood pressure and inflammation in mice fed NCD, HFD or HFD with bilberries: Figure S1, Insulin sensitivity at 7 weeks in mice fed NCD, HFD or HFD with bilberries. Figure S2, Insulin sensitivity at 11–12 weeks in mice fed NCD, HFD or HFD with bilberries. Figure S3, Glucose tolerance in mice fed NCD, HFD or HFD with bilberries. Figure S4, The net-effect of serum pro-inflammatory cytokines in mice fed NCD, HFD or HFD with bilberries. Figure S5, Correlation of inflammatory cells and adiponectin with blood pressure. Table S3, Blood lipids of mice fed NCD, HFD or HFD with bilberries. Table S4, Serum levels of pro- and anti-inflammatory cytokines in mice fed NCD, HFD or HFD with bilberries. Table S5, Systolic blood pressure and heart rate in mice NCD, HFD or HFD with bilberries. Table S6, Correlation of inflammatory cells with cytokines and adipokines. (PDF) [file pone.0114790.s001.pdf]

Panel S1. Supporting phenotypic characteristics.

Panel S1 FIGURES

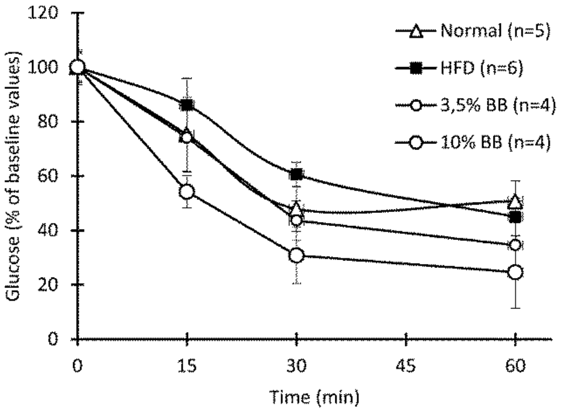

Figure S1. Insulin sensitivity at 7 weeks in mice fed NCD, HFD or HFD with bilberries. The effect of bilberries (3.5 and 10% BB w/w) on insulin sensitivity was measured in a pilot experiment at week 7 using a high-fat diet (HFD) C57BL mouse model.

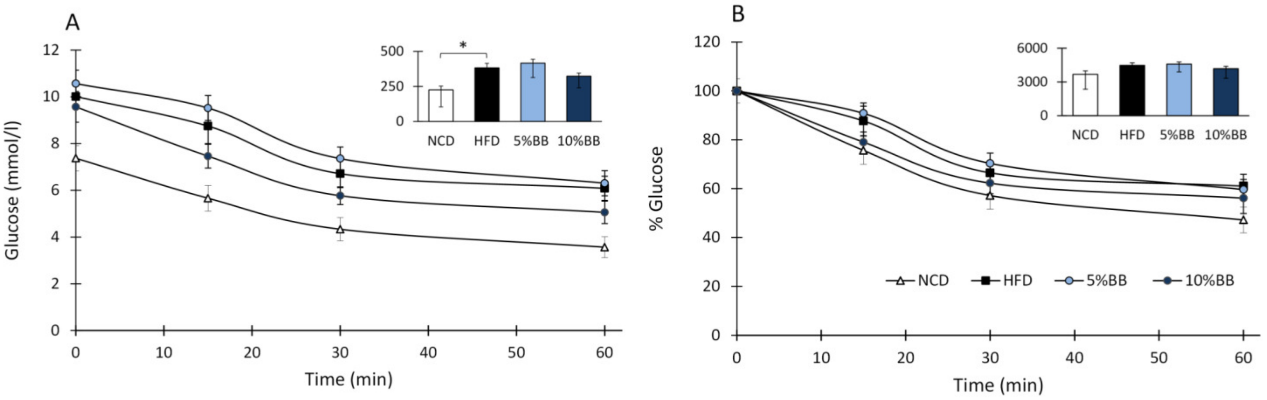

Figure S2. Insulin sensitivity at 11-12 weeks in mice fed NCD, HFD or HFD with bilberries. Intraperitoneal insulin sensitivity test (IPIST) at 11-12 weeks of mice fed with normal control diet (NCD, n = 20), high-fat diet (HFD, n = 16), 5% and 10% (w/w) bilberries in HFD (n = 16 per group). Mice were fasted and blood glucose measured after i.p. administered bolus of insulin (0.25 IU/kg weight of mice). The histograms display $AUC_{glucose}$ (mmol/l x min). Panel A shows the absolute values and panel B the values as percentage from the baseline. The bars represent SEM (curve) and SEM (top histogram) and SD (below histogram). Animals with failed injections and measurements or reflecting stress indicated by abnormally high glucose values (over 150% at 15 -30 min or over 100% at 60 min as compared to baseline) were excluded: NCD (1), HFD (3), 5%BB (8), 10%BB (3). P-values vs. the HFD fed mice: * < 0.01.

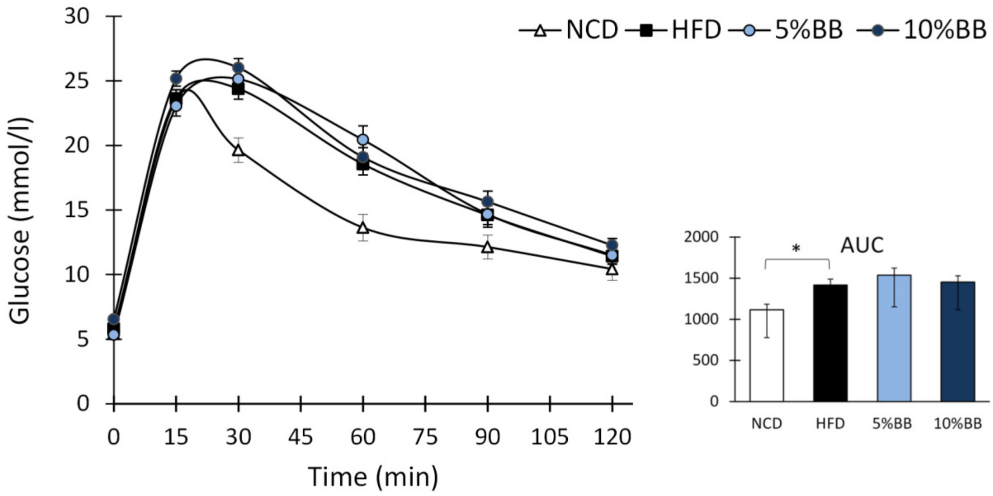

Figure S3. Glucose tolerance of mice fed with NCD, HFD or HFD with bilberries. The intraperitoneal glucose tolerance test (IPGTT) results from mice fed for 9 weeks with normal control diet (NCD, n = 23), high-fat diet (HFD, n = 24), 5% (n=21) and 10% (w/w) bilberries in HFD (n = 19 per group). Blood glucose was measured after 12-14 hour fasting (0 min) and 15-120 min after i.p. administered glucose (20% w/v). The histogram displays $AUC_{glucose}$ (mmol/l x min). The error bars represent SEM (curve) and SEM (above) and SD (below) the histogram. Animals with failed injections and measurements or reflecting stress indicated by abnormally high glucose values were excluded: NCD (4), HFD (4), 5%BB (2) and 10%BB (1). P-values vs. the HFD fed mice: * < 0.001.

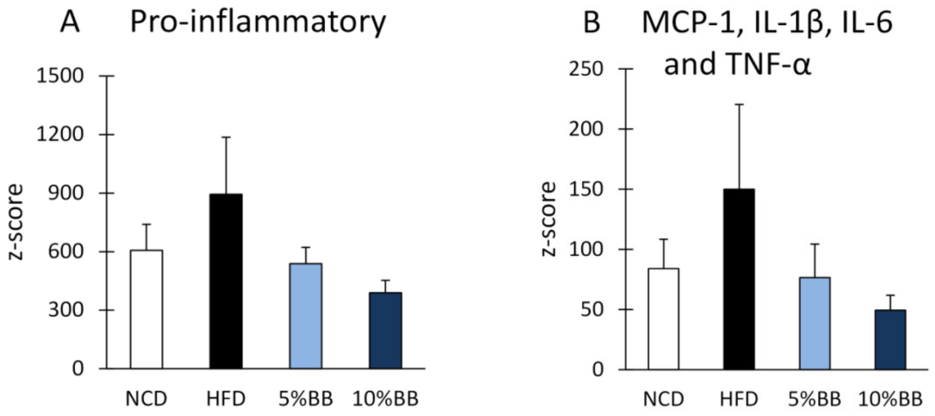

Figure S4. The net effect of serum pro-inflammatory cytokines in mice fed NCD, HFD or HFD with bilberries. The net effect of pro-inflammatory cytokines was measured in mice fed with normal control diet (NCD), high-fat diet (HFD) and 5% or 10% bilberries (BB) in HFD for 12-14 weeks. The z-score of the common pro-inflammatory cytokines IL-1α, IL-1β, IL-2, IL-6, IL-12, IL-15, IL-17, RANTES and TNF-α (A) and the most prominent obesity associated pro-inflammatory cytokines; MCP-1α, IL-1β, IL-6 and TNF-α (B). Histograms show mean values of summary z-scores of the representative cytokines and SEM (bars) of n = 8 mice per group.

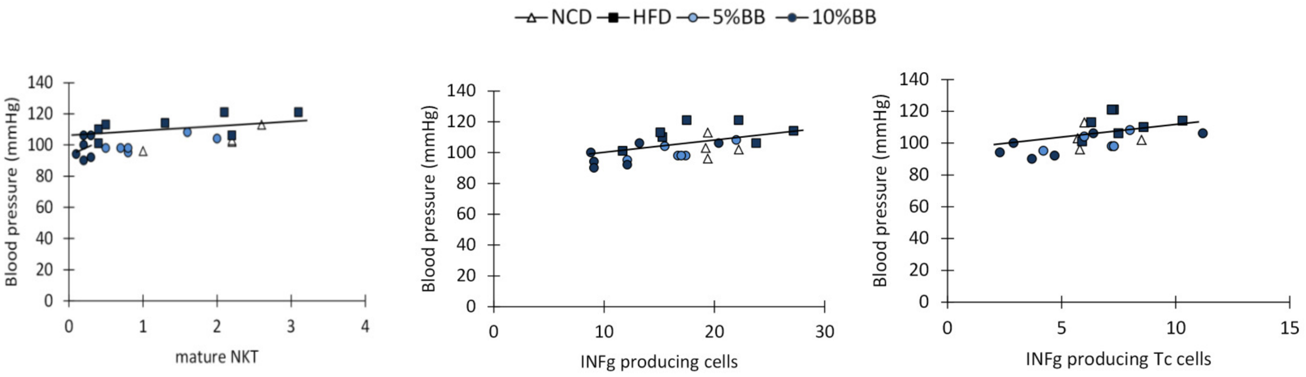

Figure S5. Correlation of inflammatory cells and adiponectin with blood pressure. The correlation of the relative number of mature NKT, INF-γ producing cells, INF-γ producing cytotoxic T cells, T helper type 1 cells and adiponectin with blood pressure was measured in mice fed with normal control diet (NCD), high-fat diet (HFD) and 5% or 10% bilberries (BB) in HFD for 12-14 weeks. Each symbol represents an individual measurement. Significant correlations indicated by a trend line (Spearman's test, $p<0.05$).

Table S3. Blood lipids of mice fed with NCD, HFD, or HFD with 5 or 10% bilberries (w/w). Total cholesterol (tCHOL), HDL and LDL cholesterol, triglycerides (TRIG) and free fatty acids (FFA), mmol/L, mean values ± SEM.

Diet	tCHOL	HDL	LDL	TRIG	FFA
**NCD**	2.5 ± 0.1	2.3 ± 0.1	0.4 ± 0.04	0.9 ± 0.8	1.0 ± 0.1
**HFD**	3.4 ± 0.2*	3.1 ± 0.2*	0.5 ± 0.04	0.8 ± 0.1	1.3 ± 0.1
**HFD +5%BB**	3.9 ± 0.2	3.5 ± 0.2	0.5 ± 0.04	0.9 ± 0.1	1.3 ± 0.1
**HFD +10%BB**	4.1 ± 0.2	3.7 ± 0.2	0.5 ± 0.05	1.0 ± 0.1	1.0 ± 0.1

a) One-way ANOVA followed by LSD post hoc test and Bonferroni's correction between; NCD and HFD, 5% BB or 10% BB and HFD, *p<0.05.
The number of replicates was in NCD (12/14), HFD (17/18), HFD with 5% (9/19) and 10% bilberries (11/13).
The values for total and HDL and LDL deviating over 20% from the mean were excluded from the results; NCD (2), HFD (1), 5%BB (1), 10%BB (2).

Table S4. Serum levels of pro- and anti-inflammatory cytokines in mice fed NCD, HFD or HFD with bilberries. The serum levels of pro-inflammatory and anti-inflammatory cytokines were measured in mice fed with normal control diet (NCD), high-fat diet (HFD) and 5% or 10% bilberries (BB) in HFD for 12-14 weeks. Values represent means ± SEM of n = 3-15 mice per group.

**Cytokines (pg/ml)**	**NCD**	**HFD**	**5% BB in HFD**	**10% BB in HFD**
IL1α	29.8 ± 6.9	107 ± 46.5	105 ± 46	ND
**IL1β**	37.8 ± 8.8	80.3 ± 34.9	**16.1 ± 0.8**	**16.5 ± 1.5**
**IL2**	20.8 ± 8.1	44.0 ± 19.2	**3.9 ± 1.0**	9.9 ± 6.6
IL5	16.6 ± 3.0	19.8 ± 3.3	15.2 ± 2.1	15.9 ± 3.4
**IL6**	28.3 ± 9.1	29.8 ± 7.8	**12.3 ± 2.9**	42.1 ± 14.9
**IL7**	21.7 ± 4.0	39.4 ± 14.4	**7.7 ± 1.8**	10.3 ± 3.7
IL9	456.7 ± 146.5	745.9 ± 310.7	323.4 ± 98.7	155.9 ± 33.4
IL12	230 ± 53.9	376 ± 104	352 ± 64	230 ± 55
**IL13**	17.2 ± 4.9	**112.3 ± 49.9**	53.7 ± 46.0	ND
IL15	218.6 ± 62.0	649.9 ± 245.3	ND	ND
IL17	29.5 ± 3.0	40.4 ± 4.9	32.4 ± 3.3	33.3 ± 4.1
**G-CSF**	518 ± 131.0	669 ± 141	797 ± 226	**1109 ± 132**
**GM-CSF**	311 ± 88.9	525 ± 162	450 ± 110	**139 ± 47**
INFγ	16.0 ± 4.1	39.8 ± 15.4	ND	ND
IP10	3319 ± 486	3352 ± 305	2821 ± 472	3700 ± 238
KC	22.8 ± 7.4	47.8 ± 12.3	44.3 ± 19.7	44.7 ± 11.6
**MCP1(CCL2)**	144 ± 44.0	248 ± 115	**37.1 ± 7.6**[a]	**27.2 ± 3.8**[a]
MIP1α	226 ± 72.6	343 ± 156	84.7 ± 13.9	48.5 ± 10.6
RANTES	26.5 ± 2.4	28.5 ± 1.6	30.6 ± 1.6	26.1 ± 1.7
tPAI1	989 ± 243	1156 ± 112	1229 ± 277	876 ± 81
**TNFα**	17.1 ± 3.8	22.1 ± 7.5	9.1 ± 0.5	**8.7 ± 1.4**

ND – not detected values

a) Significantly different from HFD ($p < 0.05$, Mann Whitney U-Test with Bonferroni's correction; **in bold** the trend for difference from NCD (HFD) or from HFD (5% BB or 10% BB in HFD ($p < 0.1$)

Table S5. Systolic blood pressure and heart rate in mice NCD, HFD or HFD with bilberries. Systolic blood pressure (mmHg) was measured by the non-invasive Tail Cuffs photoelectric method 7-8 and 11-12 weeks after the start of the diets.

	After 7-8 weeks			After 11-12 weeks		
**Diet**	**n [a]**	**SBP (mmHg)**	**HR (bpm)**	**n [a]**	**SBP (mmHg)**	**HR (bpm)**
**NCD**	12	95 ± 2	403 ± 5	12	101 ± 2	402 ± 8
**HFD**	12	99 ± 2	404 ± 5	12	109 ± 2[b]	406 ± 5
**HFD +5%BB**	10	98 ± 4	390 ± 7	10	100 ± 2[b]	407 ± 5
**HFD +10%BB**	13	92 ± 2[b]	393 ± 4	14	96 ± 2[c]	403± 3

a) Mean ± SEM from more than 5 repeated measurements per time point per animal.
b) Significantly different from NCD (HFD) or from HFD (5%BB in HFD) ($p<0.05$, Mann Whitney U-Test with Bonferroni´s correction)
c) Significantly different from HFD (10%BB in HFD) ($p<0.001$, Mann Whitney U-Test with Bonferroni´s correction).

Table S6. Correlation of inflammatory cells with cytokines and adipokines. The correlations of the relative number of T cells, Th cells types 1, 2 and 17, cytotoxic T cells (Tc), interferon gamma (INF-γ^+) producing cells, natural killer type T cells (NKT) and mature NKT cells (m NKT) with the levels of cytokines and adipokines in mice fed with normal control diet (NCD), high-fat diet (HFD), or 5% or 10% bilberries (BB) in HFD for 12-14 weeks. Only significant correlations with n = 7-25 mice within the tests are displayed (in parentheses the p-values, Spearman's test).

	**T cells**	**Th1**	**Th2**	**Th17**	**Tc**	**INF-γ^+**	**NKT**	**m NKT**
**G-CSF**	+0.63 (0.001)			-0.46 (0.020)		-0.53, (0.006)	-0.54, (0.006)	-0.70 (0.000)
**GM-CSF**				+0.52 (0.019)	+0.48 (0.034)	+0.48 (0.034) (Tc cells)		
**MIP-1α**	-0.45 (0.048)	+0.58 (0.007)		+0.59 (0.006)			+0.48 (0.033)	+0.60 (0.037)
**MCP-1**			-0.88 (0.004)					
**TNF-α**	-0.55 (0.004)	+0.49 (0.012)		+0.59 (0.002)		+0.45 (0.023)	+0.48 (0.015)	
**INF-γ**								+0.85 (0.007)
**IL-1α**								-0.74 (0.035)
**IL-5**		+0.57 (0.003)		+0.44 (0.027)			-0.54 (0.005)	
**IL-7**		+0.50 (0.04)			+0.57 (0.017)			
**IL-13**								-0.76 (0.004)
**IL-15**								-0.88 (0.008)
**IP-10**					-0.53 (0.006)			
**Insulin**		-0.58 (0.011)						
**Resistin**								+0.49 (0.040)
