# Supplementary material for: Wild Blueberries (Vaccinium myrtillus) Alleviate Inflammation and Hypertension Associated with Developing Obesity in Mice Fed with a High-Fat Diet
Source: PLoS One. 2014 Dec 12;9(12):e114790. doi: 10.1371/journal.pone.0114790 (PMC4264776; doi:10.1371/journal.pone.0114790)
Supplement: S1 Table — Details of the diets. Contents of the diets as per weight and energy, profiles of anthocyanins, lipids and salt. (PDF) [file pone.0114790.s002.pdf]

Table S1: Details of the diets. Detailed ingredients and profiles of salt, lipid and anthocyanins in the diets.

**DIET**	**NCD**		**HFD**		**5%BB in HFD**		**10%BB in HFD**	
**Macronutrients**	**g**	**% kcal**	**g**	**% kcal**	**g**	**% kcal**	**g**	**% kcal**
Protein	19.2	20	23.7	20	23.5	21	21.2	19
Carbohydrates	67.3	70	41.4	35	39.9	33	41.8	38
Fat	4.3	10	23.6	45	23.5	46	21.2	43
Total	90.8	100	88.7	100	86.9	100	84.2	100
**Ingredients**	**g**	**kcal**	**g**	**kcal**	**g**	**kcal**	**g**	**kcal**
Casein (80 Mesh)	190	758	233	932	232	928	210	839
L-Cystine	3	11	3	14	3	14	3	13
Corn starch	299	1194	85	339	84	338	77	305
Maltodextrin	33	133	117	466	116	464	105	419
Sucrose	332	1327	201	806	172	688	181	725
Cellulose (BW 200)	47		58		44		52	
Soybean oil	24	213	29	262	29	261	26	236
Lard	19	171	207	1862	206	1854	186	1676
Minerals (S10026)	9		12		12		11	
CaPO (diphasic)	12		15		15		14	
Calcium carbonate	5		6		6		5	
Potassium citrate	16		19		16		17	
Choline bitartrate	2		2		2		2	
Vitamins (V10001)	9	38	12	47	12	46	11	42
Bilberry powder	0		0		50	105	100	303
Yellow dye	0.05		0		0.025		0	
Red Dye	0		0.05		0		0.05	
Blue Dye	0		0		0.025		0	
**Total**	**1000**	**3845**	**1000**	**4728**	**1000**	**4699**	**1000**	**4557**
**Energy (kcal/g)**		**3.85**		**4.73**		**4.70**		**4.56**
**Lipid profile** (% fat)		**NCD**		**HFD**		**5%BB in HFD**		**10%BB in HFD**
Saturated		25		36		36		36
Monounsaturated		35		45		45		45
Polyunsaturated		40		19		19		19
Cholesterol (mg/kg)		18		197		197		177
**Salt profile** (mg/g)								
NaCl		2.45		3.02		2.94		2.72
Na		1.01		1.24		1.22		1.15
**Anthocyanin profile** (percent in 5% or 10%BB):								
Delphinidin-glycosides (44/40%)						0.31		1.20
Cyanidin -glycosides (27/30%)						0.19		0.92
Petunidin-glycosides (15/13%)						0.11		0.40
Peonidin -glycosides (3/4%)						0.03		0.13
Malvidn-glycosides (11/13%)						0.08		0.39
**Total (mg/g)**		0		0		**0.71**		**3.05**

NCD; normal control diet (D12450B) and **HFD**; high-fat diet (D12451), Research Diets, Inc. New Brunswick, NJ, USA.
