# Supplementary material for: Wild Blueberries (Vaccinium myrtillus) Alleviate Inflammation and Hypertension Associated with Developing Obesity in Mice Fed with a High-Fat Diet
Source: PLoS One. 2014 Dec 12;9(12):e114790. doi: 10.1371/journal.pone.0114790 (PMC4264776; doi:10.1371/journal.pone.0114790)
Supplement: S2 Table — Intakes of food, water and energy. Mean intakes of food and water consumed per animal, calculated values of energy and diet per animal weight at the beginning, middle and the end of the study. (PDF) [file pone.0114790.s003.pdf]

Table S2: Intakes of food, water and energy. The intakes of food, water and energy was measured in mice fed NCD, HFD, or HFD with 5 or 10% bilberries (w/w). (A) The individual values of mean values ± SEM (g/ml/day) from 2 mice per cage measured at weeks 1-2 (beginning), weeks 5-7 (middle) and weeks 11-12 (end). (B) The weight adjusted values of food and energy intake in each group and time point of study.

A

		Intake of food and water (g or ml per animal per day)						
		Beginning		Middle		End		Mean
Group	n (F/W)	Food	Water	Food	Water	Food	Water	Food
**NCD**	24/18	2.8 ± 0.1	3.1 ± 0.1	3.2 ± 0.1	4.1 ± 0.2	3.0 ± 0.1	4.7 ± 0.3	3.0 ± 0.1
**HFD**	22/16	2.6 ± 0.1[a]	3.3 ± 0.1	2.7 ± 0.1[a]	3.7 ± 0.2	2.4 ± 0.1[a]	3.9 ± 0.2[a]	2.5 ± 0.1[a]
**5%BB**	18/12	2.7 ± 0.1	3.4 ± 0.2	2.8 ± 0.1	3.9 ± 0.3	2.7 ± 0.2	3.8 ± 0.3	2.7 ± 0.1
**10%BB**	20/20	2.6 ± 0.1	3.1 ± 0.2	2.3 ± 0.0[a]	3.7 ± 0.3	2.2 ± 0.1	3.2 ± 0.2[a]	2.3 ± 0.1[a]

B

	Intakes of energy (kcal) and food (g) per g of body weight per day					
	Beginning		Middle		End	
Group	Energy/g	Diet/g	Energy/g	Diet/g	Energy/g	Diet/g
**NCD**	0.64 ± 0.02	0.17 ± 0.01	0.50 ± 0.01	0.13 ± 0.00	0.43 ± 0.01	0.11 ± 0.00
**HFD**	0.76 ± 0.03	0.16 ± 0.01	0.49 ± 0.01	0.11 ± 0.00[b]	0.40 ± 0.01[b]	0.09 ± 0.00
**5%BB**	0.70 ± 0.03	0.15 ± 0.01	0.50 ± 0.01	0.11 ± 0.00	0.43 ± 0.02	0.09 ± 0.00
**10%BB**	0.64 ± 0.02	0.14 ± 0.00	0.45 ± 0.01[b]	0.10 ± 0.00[a]	0.41 ± 0.02	0.09 ± 0.00

Mann Whitney U-Test with Bonferroni's correction was used to test the significance of the differences of the HFD group from NCD and 5%BB or 10%BB in HFD groups a) $P < 0.05$ or b) $P < 0.001$.

In addition to the above, a reduction in water intake in HFD vs. NCD and BB fed mice vs HFD at weeks 5-6 and weeks 10-11 (n=6 per group) was observed using the indirect calorimetry system (data not shown).
